# A common neural signature of brain injury in concussion and subconcussion

**DOI:** 10.1126/sciadv.aau3460

**Published:** 2019-08-07

**Authors:** Adnan A. Hirad, Jeffrey J. Bazarian, Kian Merchant-Borna, Frank E. Garcea, Sarah Heilbronner, David Paul, Eric B. Hintz, Edwin van Wijngaarden, Giovanni Schifitto, David W. Wright, Tamara R. Espinoza, Bradford Z. Mahon

**Affiliations:** 1 Department of Emergency Medicine, University of Rochester Medical Center, Rochester, NY 14642, USA.; 2 Department of Clinical and Translational Science, University of Rochester Medical Center, Rochester, NY 14642, USA.; 3 Center for Visual Sciences, University of Rochester, Rochester, NY 14642, USA.; 4 Moss Rehabilitation Research Institute, Elkins Park, PA 19027, USA.; 5 Department of Pharmacology and Physiology, University of Rochester Medical Center, Rochester, NY 14642, USA.; 6 Department of Neuroscience, University of Minnesota, Minneapolis, MN 55455, USA.; 7 Department of Neurosurgery, University of Rochester Medical Center, Rochester, NY 14642, USA.; 8 Division of Neurosurgery, San Antonio Military Medical Center, San Antonio, TX 78234, USA.; 9 Department of Public Health Sciences, University of Rochester Medical Center, Rochester, NY 14642, USA.; 10 Department of Neurology, University of Rochester Medical Center, Rochester, NY 14642, USA.; 11 Department of Emergency Medicine, Emory University School of Medicine, Atlanta, GA 30322, USA.; 12 Department of Psychology, Carnegie Mellon University, Pittsburgh, PA 15213, USA.; 13 Carnegie Mellon Neuroscience Institute, Carnegie Mellon University, Pittsburgh, PA 15213, USA.; 14 Department of Neurological Surgery, University of Pittsburgh, Pittsburgh, PA 15213, USA.

## Abstract

The midbrain is biomechanically susceptible to force loading from repetitive subconcussive head impacts (RSHI), is a site of tauopathy in chronic traumatic encephalopathy (CTE), and regulates functions (e.g., eye movements) often disrupted in concussion. In a prospective longitudinal design, we demonstrate there are reductions in midbrain white matter integrity due to a single season of collegiate football, and that the amount of reduction in midbrain white matter integrity is related to the amount of rotational acceleration to which players’ brains are exposed. We then replicate the observation of reduced midbrain white matter integrity in a retrospective cohort of individuals with frank concussion, and further show that variance in white matter integrity is correlated with levels of serum-based tau, a marker of blood-brain barrier disruption. These findings mean that noninvasive structural MRI of the midbrain is a succinct index of both clinically silent white matter injury as well as frank concussion.

## Teaser

Clinically silent brain injury in contact sports athletes can be detected by noninvasive MRI.

## INTRODUCTION

The current definition of concussion or mild traumatic brain injury (mTBI) is based on a consensus statement issued at the 4th International Conference on Concussion in Sport (ICCS) (*1*): “the rapid onset of short-lived impairment of neurological function that resolves spontaneously…caused either by a direct blow to the head, face, neck or elsewhere on the body with an ‘impulsive’ force transmitted to the head.” There is tension between that consensus statement and scientific studies demonstrating that repetitive subconcussive head impacts (RSHIs) sustained in the setting of American football without frank concussion can produce acute neurophysiological changes (*2*, *3*) and are thought to be, in the long term, associated with neurodegenerative diseases such as chronic traumatic encephalopathy (CTE) (*4*–*10*). The acute brain pathology produced by the forces in mTBI and RSHI is considered to be diffuse axonal injury (DAI), which affects diverse regions of the brain (*2*, *11*). Accordingly, most research into RSHI and concussion using magnetic resonance imaging (MRI)–based metrics has focused on whole-brain analyses. The result is that we currently lack a hypothesis-driven noninvasive approach to assess the occurrence, recovery, and chronic burden of either asymptomatic or symptomatic head injury. Here, we propose that the midbrain is a key structure that can serve as an index of injury in both clinically defined concussions and RSHI.

The brainstem is biomechanically vulnerable to injury from head hits due to its narrow dimensions and differential sensitivity to rotational loading (*12*). Finite element analysis, a quantitative method used to study stress and strain in response to an external force, has shown that the highest strains following a concussive impact in boxers and football players are concentrated in the midbrain (*13*, *14*). The brainstem (which includes the midbrain) is an important site of axonal injury in histopathological studies of mTBI performed in animals and humans (*11*). Clinically, the preponderance of oculomotor and auditory processing dysfunction among mTBI patients provides convergent evidence for involvement of the midbrain in mTBI pathology. Epidemiologically, up to 86% of mTBI patients have oculomotor dysfunction (*15*) and more than 50% of mTBI patients have auditory processing signs and symptoms. The midbrain is also involved in memory, consciousness, and autonomic function, systems prominently affected in concussion. Chronically, CTE patients show signs of tauopathy in the brainstem, especially the midbrain, in both histopathological and imaging studies (*7*, *16*, *17*). Together, these studies motivate the hypothesis that the midbrain is an epicenter of mTBI pathology. However, it remains an open empirical question whether structural changes to the midbrain are observed in RSHI and whether those same changes are present in the setting of clinically defined concussion.

Inertial loading from a concussive impact includes both linear and rotational acceleration. While linear acceleration is correlated with increased intracranial pressure and accounts for some neurological dysfunction, it is not associated with acute mental state alterations—the pathognomonic feature of mTBI (*18*). In contrast, rotational acceleration is found to account for 78% of the variance in shear stress (*19*), which plays an important role in the occurrence of DAI. Head impacts, such as those that typically occur in the setting of American football, are associated with a rapid onset of rotational forces, which cause shear waves that are transmitted throughout the brain. For this reason, it has been suggested that loss of consciousness secondary to mTBI does not occur in the absence of rotational acceleration (*20*). It has also been argued that rotational forces have a dose-dependent correlation with DAI in the brainstem in primate and porcine models of mTBI (*11*, *20*). These findings have not been demonstrated in humans—the only way to carry out such a test in humans is to prospectively record rotational forces together with noninvasive markers of the structural integrity of midbrain areas. Here, we test the empirical hypothesis that rotational loading explains variance in changes in white matter structural integrity in the midbrain, in the setting of RSHI.

The objective of this study was to test the hypotheses that (i) repetitive head hits in the absence of clinically defined concussion, as well as head hits associated with a clinically defined concussion, will both be associated with a decrease in white matter integrity in the midbrain, and (ii) in the setting of repetitive head hits in the absence of clinically defined concussion, the amount of disruption to white matter integrity in the midbrain will be related to the number of hits with high rotational acceleration. We also report preliminary evidence that changes in the white matter structure in the midbrain are related to serum-based tau levels, an index of breakdown in the blood-brain barrier and axonal injury. We note, up front, that previous studies have documented DAI in regions of the brain in addition to the midbrain (*21*, *22*), including the corpus callosum, internal capsule, superior longitudinal fasciculus, corona radiata, and thalamic radiations (*23*). The presumption of our core hypotheses is not that the midbrain is the only site of white matter injury in the brain following mild neurotrauma; rather, we test the hypothesis that the midbrain provides a reliable and sufficient index of both clinically silent and clinically diagnosed neurotrauma.

Two separate cohorts of participants were studied to evaluate these hypotheses. The first cohort (*n* = 38) consisted of collegiate football players who were followed with pre- and postseason MRI while wearing helmet-worn accelerometers for all practice and play. The second cohort consisted of patients with clinically defined concussion or mTBI (*n* = 29) who were compared to age-matched controls (*n* = 58). Hypothesis testing focused on the corticospinal tract (CST) at the midbrain level because this pathway carries projecting fibers between the midbrain and the cortex, especially those related to oculomotor function. To ensure rigor and reproducibility of all findings, standard atlases were used to define all regions of interest (ROIs) using widely available analysis pipelines. White matter structural integrity was measured using fractional anisotropy (FA), which has been widely used to index the structural integrity of major white matter pathways in the brain (*21*).

## METHODS

### Participants

#### RSHI cohort

Participants were 42 male football players on the University of Rochester football team [National Collegiate Athletic Association (NCAA) division III], enrolled over the course of three seasons (2011, 2012, and 2013). Three participants were lost to follow-up, and one participant lacked field map correction scans, yielding 38 complete datasets (mean age, 19.8; median, 20). All of the players sustained repetitive head impacts across the season, but only 2 of the 38 sustained clinically defined concussion/mTBI. For all participants in the RSHI cohort, MRI scanning was carried out 2 weeks before the start of the season and within 1 week after completion of the season. To record the inertial loading sustained by the players’ brains throughout the season of play, each player wore a helmet-mounted accelerometer that measured linear and rotational acceleration throughout all practices and games. Full details regarding helmet accelerometers to measure head impacts have been previously described (*24*), including the derivation of rotational acceleration via the accelerometer data. In brief, each athlete was outfitted with a Riddell Revolution IQ helmet (Riddell Corporation, Elyria, OH) equipped with Head Impact Telemetry System (HITS) encoders (Simbex LLC, Lebanon, NH) for the duration of the season, including all practices and games. The University of Rochester Institutional Review Board approved this study, and written informed consent was obtained from all participants.

#### mTBI cohort

We conducted retrospective analyses on 29 mTBI patients [15 males and 14 females (mean age, 19.5; median age, 19)] and 58 matched controls [44 males and 17 females (mean age, 21.6; median age, 21)]. The individuals diagnosed with concussion are a subset of a broader group of NCAA contact sport athletes at the University of Rochester and the Rochester Institute of Technology who were monitored for concussion. Between 2009 and 2014, 632 NCAA division I and III collegiate contact sport athletes underwent plasma sampling and cognitive testing before the sports season and were followed prospectively for a diagnosis of mTBI. mTBI was defined as an injury witnessed by an on-field certified athletic trainer and meeting the definition of concussion as defined by the Sport Concussion Assessment Tool 2. For the concussed participants in the mTBI cohort, diffusion tensor imaging (DTI) scans and blood samples (for measures of serum-based tau) were collected within 72 hours after injury (and at the same time). We note that the participants enrolled in the mTBI cohort were not wearing accelerometers, and therefore, information about the head hits they sustained is not available for analysis.

Sixteen of the 58 controls took part in various athletic programs at the University of Rochester, but none of those 16 participated in contact sports. That initial group of 16 controls was later supplemented with an additional 42 participants recruited through other studies that matched the critical cohort on age and gender, always using the same DTI acquisition parameters. None of the controls had a history of mTBI.

### General procedures and MRI acquisition parameters

Participants were tested on a Siemens 3T Tim Trio scanner using a 32-channel head coil located at the Rochester Center for Brain Imaging (since renamed to “Center for Advanced Brain Imaging and Neurophysiology”). High-resolution structural T1 contrast images were acquired using a magnetization-prepared rapid gradient echo (MP-RAGE) pulse sequence at the start of each participant’s first scanning session [repetition time (TR) = 2530, echo time (TE) = 3.44 ms, flip angle = 7°, field of view (FOV) = 256 mm, matrix = 256 × 256, 1 mm × 1 mm × 1 mm sagittal left-to-right slices] (*25*). DTI was acquired using a single-shot spin-echo echoplanar imaging (SE-EPI) (60 diffusion directions with *b* = 1200 s/mm^2^, 10 images with *b* = 0 s/mm^2^, TR = 8900 ms, TE = 86 ms, FOV = 256 × 256 mm^2^, matrix = 128 × 128, voxel size = 2 mm^3^ by 2 mm^3^ by 2 mm^3^, 70 axial slices) (*25*). A double-echo gradient echo field map sequence (echo time difference = 2.46 ms, EPI dwell time = 0.75 ms) was acquired with the same resolution as the DTI sequence and was used to correct for distortion caused by B0 inhomogeneity (*25*).

### DTI preprocessing and analysis

Preprocessing of the diffusion data was performed using fsl-5.0.9 (FSL; www.fmrib.ox.ac.uk). Data were corrected for magnetic susceptibility distortions, motion, and eddy currents using the fugue and eddy packets in FSL. FSL’s brain extraction tool was used to skull-strip each participant’s diffusion-weighted and field map magnitude images. The B0 image was stripped from the diffusion-weighted image, and the field map was prepared using FSL’s field map preparation tool. Smoothing and regularization were performed using FSL’s fugue tool (www.fmrib.ox.ac.uk/fsl), and three-dimensional (3D) Gaussian smoothing was applied using sigma = 4 mm (*25*). The magnitude image was warped on the basis of this smoothing, with *y* as the warp direction, following a previous study (*26*). Eddy current correction was performed using FSL’s eddy_correct tool, which takes each volume of the diffusion-weighted image and registers it to the B0 image to correct for both eddy currents and motion. Next, the deformed magnitude image was registered to the B0 image using FSL’s linear image registration tool. The resulting transformation matrix was then applied to the prepared field map. Last, distortions were removed from the diffusion-weighted image using the registered field map, with FSL’s fugue tool. Intensity correction was applied to this unwarping. Upon completion of preprocessing, FSL’s DTIFIT tool was used to reconstruct the diffusion tensors. DTIFIT uses linear regression to fit a diffusion tensor model at each voxel of the preprocessed diffusion image; this results in FA, mean diffusivity, radial diffusivity, and axial diffusivity maps for each participant. Group analyses were carried out in specific ROIs (see below) by nonlinearly registering each participant’s native-space FA map to Montreal Neurological Institute (MNI) space (www.fmrib.ox.ac.uk/fsl/data/FMRIB58_FA). Following prior studies in the literature (*21*), we used FA values for hypothesis testing.

### ROI definition

To isolate the midbrain region of the CST, we multiplied a whole-brain image containing a mask of the entire midbrain with an atlas-defined mask of the CST, separately for the left and right hemispheres. The midbrain region was obtained using a standard space atlas (MNI152 T1 1mm) (*27*), and the CST map was obtained from an atlas of white matter regions in standard space (MNI152 T1 1mm) (*28*). We identified the voxels that represented the intersection of the two masks, which resulted in an objective (i.e., hands-off) definition of the CST in the midbrain. The resulting CST ROI for each participant was used to extract the average FA values from the FA map in standard (MNI) space. A schematic illustration of these steps is represented in fig. S1. The advantage of this pipeline for defining midbrain ROIs is that it does not require hand-drawn ROIs and is thus an objective means to define key regions; this ensures generalizability and replicability of the core findings.

### Relating the spatial pattern of hits across the head to hemispheric asymmetries in white matter damage

To anticipate an unpredicted finding that emerged across both cohorts, we found that the right hemisphere exhibits differential white matter injury compared to the left hemisphere. We used support vector regression (SVR), as implemented in LibSVM for MATLAB (*29*), to test for a relation between the spatial pattern of hits across the head and hemispheric asymmetries in white matter changes. This analysis was carried out over the RSHI cohort (*n* = 38), as that was the cohort for which we had accelerometer data. A laterality index was calculated for each participant: ((Right_Post-Season_ – Right_Pre-Season_) – (Left_Post-Season_ – Left_Pre-Season_))/(|Right_Post-Season_ – Right_Pre-Season_| + |Left_Post-Season_ – Left_Pre-Season_|) [see (*30*) for review and discussion]. The laterality index scales between −1 and 1 and represents the (*Y*) values to be predicted in the SVR model. The accelerometer output includes, for each registered hit, the azimuth (360° longitude) and elevation (180° latitude) of the impact: 0° azimuth is referenced to the back of the head, +90° azimuth is referenced to the right side of the head, −90° azimuth is referenced to the left side of the head, −90° elevation is pointing to the ground, and +90° elevation is pointing up. A 3D histogram of the cumulative number of hits in equally spaced 10° bins of azimuth and elevation was calculated for each participant (36 bins for azimuth; 18 bins for elevation). We refer to the 648 locations or bins containing the total number of impacts sustained for each player as the “spatial fingerprint” of hits for that player; those data were converted to a vector (length, 648) and normalized to have sum = 1 for all players. SVR was carried out using a linear kernel and with 20 support vectors (nu-SVR within LibSVM) using 38 folds. On each data fold, the SVR model was trained to map laterality indices to the spatial fingerprints for *n* − 1 subjects; the model was then tested by providing the *n*th participant’s spatial fingerprint or feature vector and having the model generate/predicted the laterality index. The squared correlation coefficient, between the model-based predicted and the observed laterality indices, was tested for significance in two ways. First, it was compared to the standard distribution (e.g., the critical *r*^2^ for 37 degrees of freedom for an α of 0.01 is 0.17). Second, 100,000 permutation tests were run; for each permutation, laterality indices and feature vectors were randomly shuffled, and then 38-fold cross-validation was carried out. The *r*^2^ value between SVR-predicted laterality indices and the ground truth was calculated for each permutation test, and the results were plotted as the null distribution. To visualize the results of the SVR analyses, feature weights from the SVR model for each azimuth-elevation bin were correlated across participants with observed laterality indices. The results are displayed as vectors at each location/bin of azimuth and elevation.

### Blood collection and tau assays

Venous blood was collected in a nonfasting state in lavender top EDTA tubes and placed on ice until processed. All blood was centrifuged less than 60 min from the time of blood draw, at 4°C at 3000 rpm for 10 min; plasma was isolated, and samples were stored in a −80°C freezer until batch assayed, blinded to all other study measures. Analyses were carried out using a proprietary single-molecule sensitive technology (*31*, *32*). Briefly, plasma levels of tau were measured using the single-molecule enzyme-linked immunoarray (Simoa) method. A combination of monoclonal antibodies was used, where a subset of those antibodies detects tau by targeting the mid-region, while another subset is used for detection against the N terminus. This method provides molecular-level sensitivity while minimizing sample use and error levels.

## RESULTS

### Accelerometer analysis

**Fig. 1:**
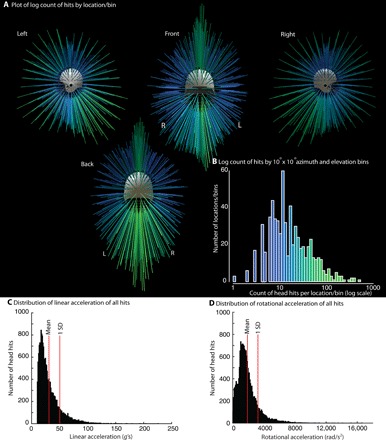
Visualization of spatial distribution of head hits in 38 collegiate football players (RSHI cohort) in a season of play. (**A**) Plot of log count of hits by location/bin. Azimuth (longitude) and elevation (latitude) were binned into 10° square bins (36 bins for 360 azimuth and 18 bins for 180° elevation). At each location/bin, the total number of hits was counted across all 38 players in the RSHI cohort. Because the distribution of hit count was strongly right skewed (higher counts for fewer number of hits per location/bin), the log_10_ of the count data was computed. The results are displayed in (A) as vectors, where the color and length of each vector scale by the log of the count of hits at that location. (**B**) Log count of hits by 10° × 10° azimuth and elevation bins. The histogram plots (*y* axis) the number of locations/bins at which (*x* axis) different numbers of hits were observed. Coloring on the histogram serves as a color scale for the data displayed in (A). (**C**) Histogram of distribution of linear acceleration for all hits. (**D**) Histogram of distribution of rotational acceleration for all hits. An interactive model of the data shown in (A) can be found at www.openbrainproject.org/tbi.

A total of 19,128 head impacts (hits) were sustained across 38 players (Fig. 1 and table S1). Of all the hits, 59% (11,334) were sustained in practice and 37% (7022) in competition. The remaining 4% of the hits were sustained in other settings such as scrimmages and meetings. In games, players sustained hits with a median rotational acceleration of 1631.7 rad/s^2^ (mean = 1947.5 rad/s^2^); the median rotational acceleration for hits sustained during practice was 1585.5 rad/s^2^ (mean = 1817.5 rad/s^2^). For linear acceleration, a similar pattern was observed with numerically higher median and mean linear acceleration during games (median = 25.1*g*, mean = 31.52*g*) than during practice (median = 24.9*g*, mean = 30.1*g*). Figure 1 plots the (log) count of hits across the RSHI at each location/bin on the head and shows that higher counts are associated with the mid-sagittal plane.

### RSHI cohort: Midbrain white matter integrity

**Fig. 2:**
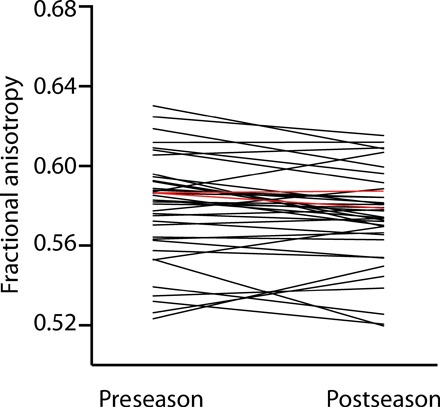
White matter integrity is reduced postseason compared to preseason in the RSHI cohort. FA in the right CST midbrain ROI was significantly reduced postseason compared to preseason. The red data points/lines correspond to the two players (of the group of 38) who sustained a frank concussion.

To test the hypothesis that there is a reduction in white matter integrity in the midbrain after a season of collegiate football, we compared pre- and postseason measures of FA from midbrain cortical spinal tract ROIs. Statistical testing used parametric (paired *t* test, two-tailed) and nonparametric tests (Wilcoxon matched-pairs rank test, two-tailed). In the right midbrain, FA was reduced postseason compared to preseason (*t* = 2.33, *P* < 0.03; Wilcoxon matched-pairs rank test, *P* < 0.02; Fig. 2). The left midbrain showed a trend in the same direction (*t* = 1.69, *P* = 0.099; Wilcoxon matched-pairs rank test, *P* = 0.10). When the analysis excluded the two players who sustained a clinically defined concussion, the difference in the right hemisphere remained significant (*t* = 2.24, *P* < 0.032; Wilcoxon matched-pairs rank test, *P* < 0.03).

### RSHI cohort: Relation between head hits and measures of white matter integrity

**Fig. 3:**
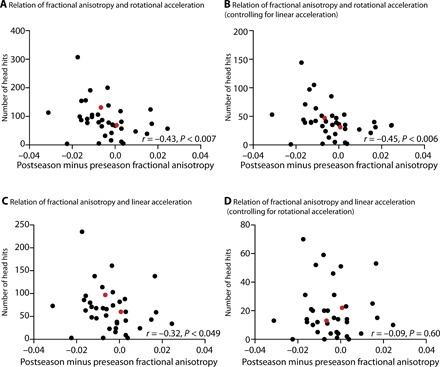
Correlation between head hits with changes in FA. In all plots, black circles indicate individuals without clinically diagnosed mTBI and red circles indicate the two individuals who suffered a concussion between the pre- and postseason MRI. (**A**) The scatter plot shows the relation between changes in FA in the right midbrain and the number of head impacts with rotational acceleration equal to or greater than 1 SD above the group mean. The direction of the relation indicates that more trauma is associated with greater reductions in the structural integrity of white matter. This relation remained when excluding the two participants who suffered a clinically defined concussion (*r* = −0.42, *P* < 0.012). (**B**) The relation between rotational acceleration and changes in FA holds when controlling for linear acceleration by restricting the analysis to hits that exceed the threshold for rotational acceleration but do not exceed the threshold for linear acceleration. This relation remained when excluding the two participants who suffered a clinically defined concussion (*r* = −0.44, *P* < 0.008). (**C**) The number of head hits with linear acceleration greater than 1 SD above the mean is negatively correlated with the change (postseason minus preseason) in FA in the midbrain. This relation, however, was not significant when excluding the two participants who suffered a clinically defined concussion (*r* = −0.32, *P* = 0.06). (**D**) The relation between linear acceleration and changes in FA goes away when controlling for rotational acceleration by restricting the analysis to hits that exceed the threshold for linear acceleration but do not exceed the threshold for rotational acceleration. This lack of a relation between linear acceleration and changes in DTI remained absent when excluding the two participants who suffered a clinically defined concussion (*r* = −0.11, *P* = 0.51).

If the reduced structural integrity of midbrain white matter is related to head hits, rather than nonspecific variables associated with playing football, then there should be a relation between the amount of structural degradation in the midbrain and the amount of head trauma each player sustained. To test for a link between changes in midbrain structural integrity and head hits, we correlated (Spearman) head hits (as measured by the helmet-worn accelerometers) with changes in FA across the cohort (*n* = 38). To threshold the force parameters in an objective manner, we used the number of impacts with rotational acceleration or linear acceleration 1 SD above the means for each type of inertial loading (calculated across the cohort). Those thresholds corresponded to a linear acceleration of 50.7*g* and rotational acceleration of 2782 rad/s^2^. The number of impacts with suprathreshold rotational acceleration was inversely correlated with changes in FA (*r* = −0.43, *P* < 0.008; Fig. 3A). The numbers of impacts with suprathreshold linear acceleration were marginally correlated with changes in FA (*r* = −0.32, *P* < 0.049; Fig. 3C). The core finding remained when we conducted the same analysis removing the two participants in the RSHI cohort who sustained a clinically diagnosed mTBI during the season. There was an inverse relation between rotational acceleration and changes in midbrain FA (*r* = −0.42, *P* < 0.01) but only a marginal relation for linear acceleration (*r* = −0.32, *P* < 0.06).

**Fig. 4:**
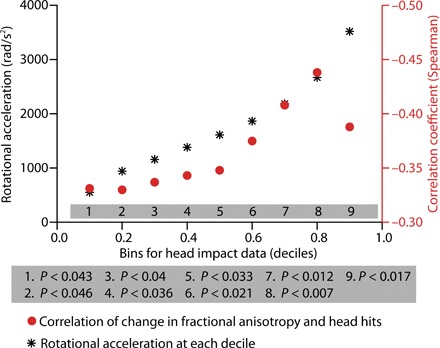
Control analyses ensured that the relation between rotational acceleration and changes in FA does not depend on thresholds. The plot shows (black stars) the rotational acceleration values corresponding to decile binning of the data. For each decile, the correlation between the number of hits (at that threshold or higher) and changes in FA was computed (filled red circles). Regardless of the threshold used, there was a significant correlation between changes in FA and number of head hits.

To ensure that the relation between rotational acceleration and changes in the structural integrity of midbrain structures was not dependent on the a priori threshold chosen for the accelerometer data (i.e., 1 SD above the mean), we repeated the analysis using different thresholds. Specifically, we binned the accelerometer measurements into deciles, calculated from the distribution of all accelerometer measurements, and then used each decile as a threshold with which to determine the correlation between the number of impacts (with force at that decile or greater) and changes in FA. The relation between head impacts and changes to FA remained significant across the full range of rotational acceleration thresholds (Fig. 4).

Last, to assess the independent effects of rotational and linear acceleration, we analyzed the number of impacts meeting the threshold for one but not the other. The number of impacts with rotational acceleration greater than 2782 rad/s^2^ and linear acceleration less than 50*g* were inversely correlated with changes in FA (*r* = −0.45, *P* < 0.006; Fig. 3B). However, hits with linear acceleration greater than 50*g* and radial acceleration less than 2782 rad/s^2^ were unrelated to changes in FA (*r* = −0.09, *P* = 0.60; Fig. 3D). These patterns remained when excluding the two players who suffered clinically defined concussion (see Fig. 3 caption for those additional statistical tests).

### mTBI cohort: Independent validation of MRI-based measures of midbrain structural integrity

**Fig. 5:**
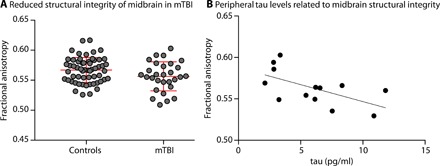
White matter structural integrity of the midbrain indexes clinically defined mTBI and relates to peripheral tau. (**A**) ROI analyses of the right CST ROI in the midbrain show significantly reduced FA in individuals diagnosed with a concussion compared to matched controls. (**B**) There is a negative correlation between peripheral tau and structural integrity of the midbrain in concussed individuals, indicating that higher levels of peripheral tau are associated with greater reductions in structural integrity of the midbrain CSTs.

The core hypothesis driving this research is that midbrain white matter integrity offers a neural sequela of repetitive subclinical concussive head impact. As an independent validation that MRI-based measures of midbrain white matter structural integrity index head trauma, we applied the same methods to a separate cohort of athletes (mTBI cohort, *n* = 29); this cohort was defined as having sustained a clinically defined concussion, and was scanned using the same MRI protocol as was the first cohort, and within 72 hours of the clinically defined concussion (see Methods). We compared the MRI-based measures of midbrain structural integrity to the same measures obtained from 58 healthy age- and sex-matched controls who did not have a history of playing contact sports. Compared to age-matched controls, there was reduced structural integrity of the right midbrain in the mTBI cohort (*t* = 2.11, *P* < 0.039; two-sample *t* test, two-tailed; Fig. 5A). Replicating the lateralized findings in the collegiate football players, FA in the left midbrain in mTBI patients was not significantly different compared to controls (*t* < 1). As noted, the mTBI cohort did not have accelerometers, and thus, impact-related metrics were not available to correlate with diffusivity measures.

### mTBI cohort: Relation between MRI-based measures of midbrain structural integrity and peripheral tau in the cohort with clinically diagnosed concussion

Blood samples were available on 13 of the 29 participants who sustained a clinically defined mTBI (blood drawn at the time of the DTI scan). If our MRI-based measure of midbrain injury is a sensitive index of disease severity in TBI, then it should be related to peripheral tau levels, which are associated with axonal injury and disruption of the blood-brain barrier. Consistent with the global hypothesis that white matter integrity of the midbrain indexes the severity of mTBI, there was an inverse correlation between peripheral tau and midbrain FA (*r* = −0.60, *P* < 0.033; Fig. 5B): Increased peripheral tau is associated with reduced structural integrity of the midbrain.

## DISCUSSION

We tested the hypothesis that MRI-based measures of midbrain white matter integrity at the level of the CSTs in collegiate football players would be decreased after a season of play compared to preseason measures. There was a reduction in FA of right midbrain structures in collegiate football players at their postseason assessment compared to their preseason assessment (Fig. 2), and this effect held when excluding the two players (of 38) who suffered a clinically defined concussion. We then tested the hypothesis that the degree of reduction in structural integrity to midbrain structures was correlated with the number of head hits the players sustained (Fig. 3). We found that reductions in midbrain white matter integrity were related to the amount of rotational (but not linear) acceleration that players’ brains sustained. Consistent with previous quantitative studies using finite element modeling, these findings indicate that rotational acceleration is the primary driver of changes in white matter structural integrity of the midbrain (*19*). Furthermore, as shown in Fig. 4, there was a consistently significant relation between the accelerometer-based measures of rotational loading and changes in FA across a broad range of thresholds on the accelerometer data. We then validated our core finding by showing reduced midbrain structural integrity in an mTBI cohort compared to age- and sex-matched controls (Fig. 5A), and finally that levels of peripheral tau were correlated with MRI-based measures of midbrain white matter structural integrity (Fig. 5B). These analyses reinforce the core inference that white matter integrity in the midbrain indexes neurotrauma common to subconcussive repetitive head hits and clinically defined concussion or mTBI.

### Explorative analyses of the relation between the spatial distribution of head hits and hemispheric asymmetries in white matter changes

**Fig. 6:**
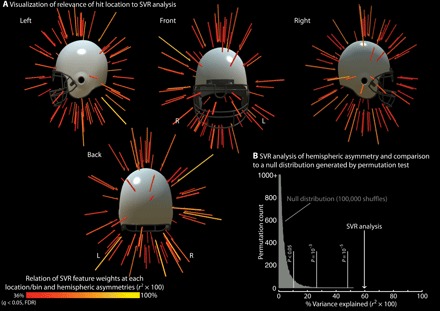
SVR analysis relating the spatial fingerprint of head impacts to hemispheric asymmetries in white matter. Each player has a “spatial fingerprint” of the distribution of hits around the head over the entire season. That pattern was used to train a linear SVR model to predict variance across participants in hemispheric asymmetries in white matter changes. (**A**) Visualization of relevance of hit location to SVR analysis. The images are a graphical representation of the SVR model relating variance across participants in the spatial distribution of hits to variance across participants in the laterality of white matter changes. This visualization of the SVR analysis was computed by correlating the variance across participants in feature weights (at each location/bin) with the variance across participants in hemispheric asymmetries in white matter changes. The results are plotted as vectors that are scaled in length and color by variance explained (Bonferroni thresholded at 05/648 = 0.00007, or a critical *r*^2^ of 0.36). (**B**) SVR analysis of hemispheric asymmetry and comparison. Using an *n* − 1 cross-validation approach, a linear SVR model captures 60% of the variance in hemispheric asymmetries in white matter changes. The variance explained by this analysis differs significantly from chance, as shown through permutation testing (100,000 shuffles). An interactive model of the data shown in (A) can be found at www.openbrainproject.org/tbi. FDR, false discovery rate.

An unpredicted finding that emerged across both cohorts was that white matter changes were differentially expressed in the right hemisphere. In a set of explorative analyses, we tested whether variance in hemispheric asymmetries in white matter changes across players in the RSHI cohort was related to the spatial “fingerprint” of head impacts. Figure 6B shows the results of a linear multivariate analysis (SVR) using an *n* − 1 cross-validation approach (see Methods for details). That analysis demonstrated a relation between the pattern of hits across the head and hemispheric asymmetries in white matter changes (*r*^2^ = 0.595, *P* < 0.0001). Figure 6B also plots a null distribution generated by a permutation tests of the same analysis performed over randomly shuffled data (100,000 shuffles) and indicates that the SVR-based prediction on unshuffled data is significantly different from the null distribution. Stated more plainly, the odds are less than 1 in 100,000 that the relation between the spatial pattern of hits and hemispheric asymmetry, captured by the SVR analysis, is due to chance (or would be observed in random data). As a way to visualize this analysis, we correlated variance across subjects in SVR feature weights, at each location on the head, with variance in the laterality index across players. The results are plotted (Fig. 6A) as force vectors scaled in color and length by the variance explained (*r*^2^). Comparing Fig. 6A with Fig. 1A, which plots the distribution of all hits across the head, it is clear that there is sparseness in terms of the locations on the head, where variance in SVR feature weights relates to variance in hemispheric asymmetries in white matter changes (see also discussion in the “Limitations and extensions” section).

### Pathophysiology of mTBI and subconcussive head trauma

The gold standard for detecting DAI is histochemical staining of brain white matter for amyloid-β protein (APP) (*33*, *34*). DTI represents a noninvasive method that has been validated against histological methods for axonal injury, demonstrating similar patterns of abnormalities on animal models of mTBI (*35*, *36*). It has also been extensively used in human mTBI research (*21*, *37*–*40*). Many of those studies support the association between decreased FA and mTBI across a range of experimental designs, including ROI and whole-brain analyses. Previous studies have shown that, within 2 weeks following injury, FA is decreased in the midline regions of the brain, such as the corpus callosum and the internal capsule (*40*–*43*). A significant correlation between decreased FA and neurocognitive changes has also been reported. For example, Niogi and colleagues (*40*) reported a correlation of decreased FA with increased reaction time, while Miles and colleagues (*44*) reported a negative correlation between decreased FA and cognitive domains related to executive planning and organization. However, to date, there have not been midbrain-targeted, hypothesis-driven ROI analysis of the kind we undertook in this study, and no studies to date have demonstrated that subconcussive head hits are associated with reductions in FA in the midbrain. Nonetheless, the available evidence dovetails with the results reported herein. For instance, in one study, boxers were imaged in a longitudinal design and FA values were found to decrease over time; furthermore, the decrease correlated with the number of knockouts sustained (*45*). In a study similar in design to our current study (pre-post season DTI, and players wore accelerometers), Davenport and colleagues (*46*) reported changes in FA postseason in high school football players and reported a correlation between hit metrics and changes in FA. However, those authors did not report the direction of change of the FA postseason or regional effects in reductions in white matter structural integrity. Together with those previous studies, our findings hearken back to early research on mTBI that emphasized the brain stem and midbrain, in particular, as sites of DAI and CTE pathology (*5*, *11*).

### Evaluating the contributions of rotational and linear acceleration in mTBI

We found that rotational acceleration, over and above linear acceleration, correlated more strongly and consistently with changes in white matter integrity (Fig. 3). There is sparse evidence using DTI and impact analyses to draw on as a direct precedent for this observation (*47*). However, we can draw on a rich history of studies of histologically confirmed DAI and force factor analyses delineating contributions of linear and rotational acceleration to injury burden and clinicopathology. Several studies, conducted in porcine and nonhuman primates, concluded that rotational acceleration was more important for producing shear strains required for DAI and mental state alterations (*11*, *20*, *48*). They demonstrated that DAI injury burden is much higher in the brainstem (which includes the midbrain) than any other region of the brain and that DAI in this region was required for the modeled mTBI clinical pathology. More recently, through extensive video analyses and force modeling studies of concussive episodes in National Football League (NFL) games, it was found that rotational acceleration was positively correlated with shear stress and linear acceleration positively correlated with intracranial pressure (*19*). Those studies also highlighted the concentration of concussive strains in the midbrain, as compared to other regions of the brain. Given the precedence established by those studies, we predicted that rotational acceleration would capture more variance in changes in white matter integrity than would linear acceleration in our subconcussive cohort. We found that, after controlling for rotational acceleration, the correlation between linear acceleration and FA changes was not significant (Fig. 3D). However, a cautionary note is warranted with regard to our findings; despite agreement of our data with previous works in animal models, more prospective longitudinal human studies are needed to draw a link between specific head impacts and the measured changes in brain structure and function (see also discussion in the “Limitations and extensions” section).

### Tau as an index of axonal injury and blood-brain barrier disruption

As a secondary finding, we observed a negative correlation between tau levels and post-injury FA values in individuals who experienced a clinically diagnosed concussion. That observation is limited by the low sample size (*n* = 13). Therefore, these findings stand to be replicated in a larger cohort. Nonetheless, the findings we report are the only evidence, of which we are aware, that specifically relate levels of peripheral tau to DTI-based measures of white matter integrity.

### Limitations and extensions

A limitation of the current report is that it is an observational study; the observational nature of the study means that care is required in drawing causal inferences from the data. For instance, player’s previous history of head hits may be correlated with their pattern of head hits in the season in which they were studied with MRI, and this is not something that could be controlled in the current study. Nonetheless, we provided evidence for causality in two ways: (i) by referencing each player’s postseason data to their preseason baseline, thus removing baseline differences across individuals, and (ii) by relating changes in DTI-based metrics to accelerometer-based measures of head hits, thus tying the DTI findings to neurotrauma from the season in which the players were followed. A second limitation is that we did not design our study to investigate the important issue of whether there are gender/sex differences in terms of how subconcussive and concussive force loading affects white matter integrity. Future work can make progress on this question through carefully establishing male and female cohorts in a prospective longitudinal design. A third limitation of our study is that we did not have neurocognitive data to relate to white matter changes. Current neurocognitive tools such as the proprietary computerized IMPACT test are validated on concussed participants who experience symptomatic, physiologic, and neuropsychologic effects (i.e., symptomatic mTBI). The applicability and sensitivity of these tests in subconcussive cohorts have not been established. A third limitation that has already been noted, but which bears emphasis, is that mTBI injury is distributed across other structures in addition to the midbrain, including the corpus callosum, internal capsule, superior longitudinal fasciculus, corona radiata, and thalamic radiations [for review, see (*23*)]. The goal of the current investigation was to undertake a hypothesis-driven study about the susceptibly of midbrain structures to white matter injury. This investigation sought, and accomplished, a demonstration of the sensitivity of midbrain structures for capturing relevant variance in subconcussive head hits. Our findings are agnostic as to whether other brain structures would also exhibit a similar stereotyped pattern of injury, or perhaps a different stereotyped pattern of injury. For instance, previous work (*49*) suggests that CTE may start in the frontal lobe. A fourth limitation that is important to emphasize is that the analyses (Fig. 6) relating the spatial fingerprint of head impacts to hemispheric asymmetries in white matter injury were not planned at the time of study design, as we did not a priori predict a left-right asymmetry in white matter changes. Hence, those analyses must be regarded as explorative and meriting replication. However, if future work establishes consistency in the spatial distribution of hits that drive hemispheric asymmetries in white matter injury, then it would be valuable to combine these findings with finite element analyses and magnetic resonance elastography. Last, it should also be noted that, while DAI is thought be the defining injury of mTBI, other pathologies such as cerebral microbleeds (CMB) are reported to be present in mTBI cohorts (*50*). Those pathologies will be important to document and separate from DAI using imaging modalities such as susceptibility-weighted and T2* MRI.

Our findings, together with the above-described limitations, motivate several concrete extensions for future research. First, American football has proven to be an important testing ground for advancing basic knowledge about how subconcussive and concussive head hits affect brain structure and function. A critical next step is to carry out a prospective study in which pre-post season diffusion spectrum MRI data are paired with helmet accelerometer data and longitudinal assessments of visual, oculomotor, and auditory processing. In the setting of such a study, it will be important to use psychophysical methods to assess visual, oculomotor, and auditory processing with high sensitivity and test-retest reliability. Another future innovation would be to collect intra-season MRI data on players, at staggered time points, to be able to relate the temporal and spatial fingerprints of recent versus less recent head hits to observed changes in white matter and perceptual and motor thresholds. This future work will be critical for understanding the parameters of the clinically silent brain injury that we have documented. Such an approach would also provide the empirical basis for developing new methods and technologies to infer the cumulative injury burden incurred during contact sports for each athlete. For instance, the types of future empirical and analytic directions briefly outlined here would make possible the development of algorithms that indicate when the recent history of force loading on a player’s brain matches patterns that are predictive of white matter injury in other players. In our view, an important goal is to push scientific understanding so that it is possible to use just the temporal and spatial fingerprint of head impacts to inform return to play decisions, rather than players needing to obtain an MRI or exhibit frank clinical signs and symptoms of mTBI. Our findings suggest that catching injury burden before it manifests as overt signs and symptoms will prove critical in protecting players from long-term neurologic injury.

## Supplementary Material

http://advances.sciencemag.org/cgi/content/full/5/8/eaau3460/DC1

Download PDF

## SUPPLEMENTARY MATERIALS

Supplementary material for this article is available at http://advances.sciencemag.org/cgi/content/full/5/8/eaau3460/DC1 Fig. S1. Schematic of how FA values were extracted for the CST ROI in the midbrain. Table S1. Summary statistics for head impact data for the RSHI cohort.
