# Supplementary material for: A common neural signature of brain injury in concussion and subconcussion
Source: Sci Adv. 2019 Aug 7;5(8):eaau3460. doi: 10.1126/sciadv.aau3460 (PMC6685720; doi:10.1126/sciadv.aau3460)
Supplement: http://advances.sciencemag.org/cgi/content/full/5/8/eaau3460/DC1 [file supp_5_8_eaau3460__index.html]

Science Advances | Science AdvancesAAASSearchScience AdvancesMenu

## Supplementary Materials

**This PDF file includes:**

- Fig. S1. Schematic of how FA values were extracted for the CST ROI in the midbrain.
- Table S1. Summary statistics for head impact data for the RSHI cohort.

Download PDF

**Files in this Data Supplement:**

- Adobe PDF - aau3460\_SM.pdf
