# Supplementary material for: A common neural signature of brain injury in concussion and subconcussion
Source: Sci Adv. 2019 Aug 7;5(8):eaau3460. doi: 10.1126/sciadv.aau3460 (PMC6685720; doi:10.1126/sciadv.aau3460)
Supplement: Download PDF [file aau3460_SM.pdf]

Science Advances
AAAS

advances.sciencemag.org/cgi/content/full/5/8/eaau3460/DC1

Supplementary Materials for

A common neural signature of brain injury in concussion and subconcussion

Adnan A. Hirad*, Jeffrey J. Bazarian, Kian Merchant-Borna, Frank E. Garcea, Sarah Heilbronner, David Paul, Eric B. Hintz, Edwin van Wijngaarden, Giovanni Schifitto, David W. Wright, Tamara R. Espinoza, Bradford Z. Mahon*

*Corresponding author. Email: bmahon@andrew.cmu.edu (B.Z.M.); adnan_hirad@urmc.rochester.edu (A.A.H.)

Published 7 August 2019, *Sci. Adv.* **5**, eaau3460 (2019)
DOI: 10.1126/sciadv.aau3460

This PDF file includes:

Fig. S1. Schematic of how FA values were extracted for the CST ROI in the midbrain.
Table S1. Summary statistics for head impact data for the RSHI cohort.

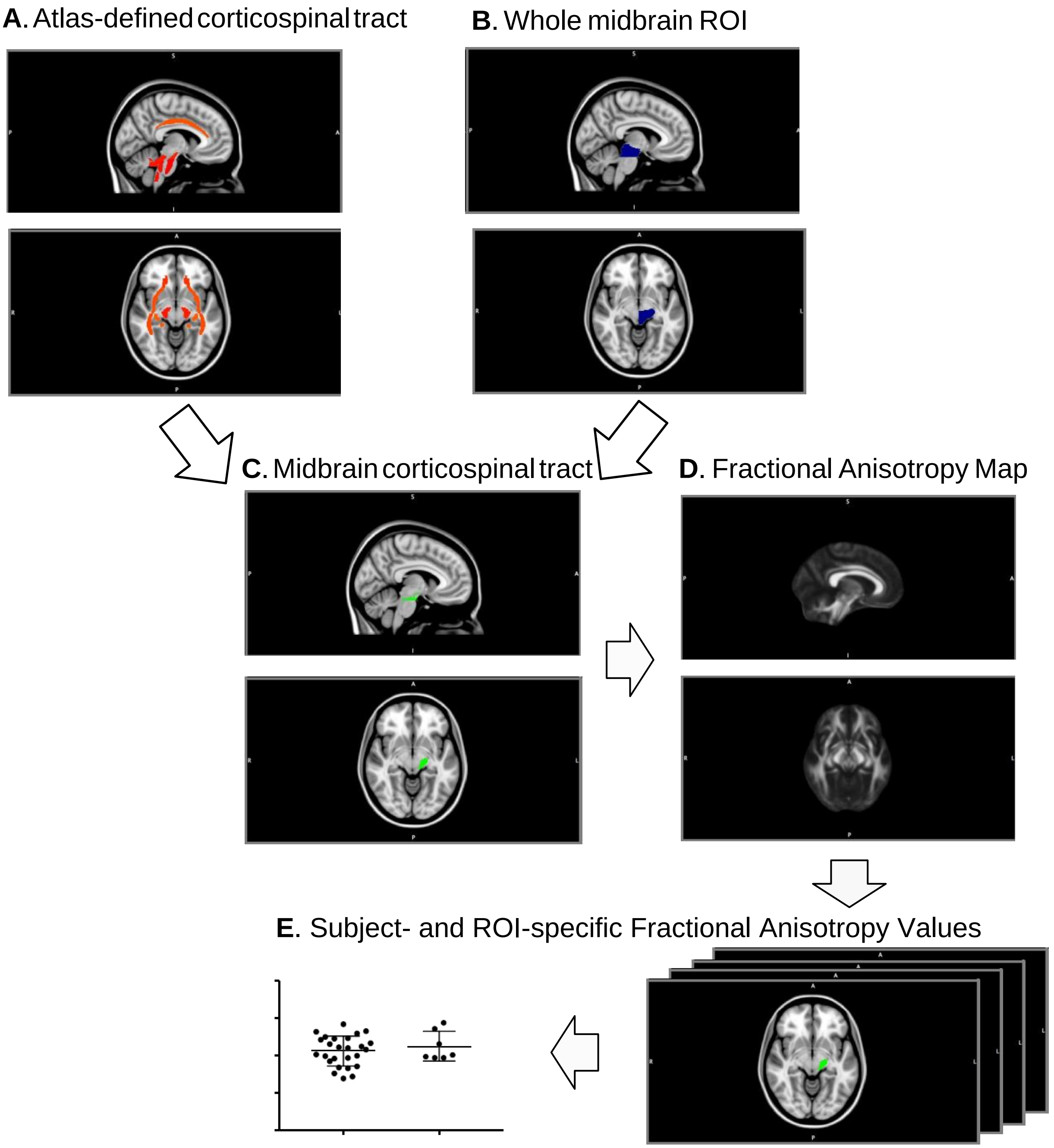

Fig. S1. Schematic of how FA values were extracted for the CST ROI in the midbrain. First, the intersection was computed between the atlas defined corticospinal tract (Panel A, colored red) and a midbrain ROI (Panel B, colored blue). The resulting midbrain ROI for the corticospinal tract (Panel C, colored green) was used to extract fractional anisotropy values from each subject's whole-brain global fractional anisotropy map (Panel D), and the results averaged over all voxels in the ROI and plotted (Panel E).

Table S1. Summary statistics for head impact data for the RSHI cohort.

Session	Rotational Acc (median)	Rotational Acc (mean)	Linear Acc (median)	Linear Acc (mean)	Numb of Hits	%Total
**Competition**	1631.7	1947.5	25.1	31.5	7022	37%
**Meeting**	1463.6	1753.7	23.1	28.5	118	1%
**Practice**	1578.7	1791.4	24.9	30.1	11334	59%
**Scrimmage**	1526.9	1878.1	24.2	30.6	654	3%
					**Total:** 19128	
